# The Mechanistic Study of Melatonin Regulation of p16Ink4a and Oct4 in Anti‐Cellular Senescence

**DOI:** 10.1111/jcmm.71321

**Published:** 2026-08-17

**Authors:** Yang Xu, Cong Zhang, Li Wang, Xiaoyu Chen

**Affiliations:** ^1^ Department of Anesthesiology General Hospital of Northern Theater Command Shenyang Liaoning China

**Keywords:** cellular senescence, melatonin, melatonin receptor

## Abstract

Melatonin is a multifunctional indole hormone with established roles in anti‐aging and tissue regeneration. Although it has been shown to modulate key senescence‐associated markers such as p16Ink4a (p16) and OCT4, the underlying molecular mechanisms remain incompletely understood. MC3T3‐E1 cells were treated with melatonin to evaluate its effects on p16 and OCT4 expression using Western blot and RT‐qPCR. Isothermal titration calorimetry (ITC) and molecular docking were employed to investigate protein–protein interactions and structural binding patterns. VDR knockdown experiments were performed to assess functional causality. In addition, osteogenic differentiation was evaluated in bone marrow‐derived mesenchymal stem cells (BMSCs) using alkaline phosphatase (ALP) and Alizarin Red S (ARS) staining. Melatonin significantly downregulated p16 and upregulated OCT4 expression without affecting cell viability. ITC analysis revealed that melatonin does not directly bind to p16 or OCT4, but both proteins interact with the vitamin D receptor (VDR) with moderate affinity. Molecular docking further supported stable binding conformations between VDR and p16/OCT4. Functionally, VDR knockdown disrupted the balance of p16 and OCT4 expression and impaired osteogenic differentiation, as evidenced by decreased ALP activity and reduced mineralized nodule formation. These findings identify VDR as a critical mediator of melatonin signalling and reveal a novel VDR‐centered regulatory axis linking senescence and stemness pathways. This mechanism provides new insights into the anti‐senescence and pro‐regenerative effects of melatonin and suggests potential therapeutic strategies for aging‐related diseases such as osteoporosis.

## 1 Introduction

Melatonin, a lipophilic indole hormone primarily synthesized and rhythmically secreted by the pineal gland, plays a crucial role in regulating various physiological processes, including circadian rhythms, metabolism, and cell survival [1]. This hormone can easily traverse the cell membrane due to its lipid‐soluble nature and has been shown to mitigate cellular senescence, a hallmark of aging. As aging progresses, melatonin secretion gradually decreases, contributing to the deterioration of cellular functions and an increase in age‐related diseases [2, 3].

Research has demonstrated the non‐toxic nature of melatonin, which, when supplemented, not only improves bone density and combats osteoporosis but also counteracts cellular senescence through various mechanisms. These mechanisms include regulation of circadian rhythms, modulation of stem cell differentiation, and protection against oxidative stress, mitochondrial dysfunction, apoptosis, and inflammation [4, 5, 6, 7, 8]. In 2017, Farr et al. proposed that targeting senescent cells expressing the p16Ink4a (p16) marker could prevent age‐related bone loss, presenting a novel strategy for managing osteoporosis [9].

Cellular senescence refers to a permanent cessation of cell division triggered by various cellular stresses. Senescent cells, often characterized by the high expression of p16, represent one of the most important markers of aging. p16 is a member of the INK4 family and primarily functions in the nucleus, inhibiting cyclin‐dependent kinases and halting the cell cycle. On the other hand, Oct4 (octamer‐binding transcription factor 4), a crucial transcription factor belonging to the POU family, has been identified as a key anti‐senescence gene. It plays an essential role in maintaining the pluripotency and self‐renewal properties of stem cells [10, 11, 12, 13]. Recent studies have shown that melatonin can reduce p16 expression in various cell types, including human odontoblasts, periodontal ligament cells, and mesenchymal stem cells [14, 15]. Additionally, melatonin significantly upregulates Oct4 expression in human embryonic stem cells and other pluripotent cell lines, such as HEK293 cells and neurogenesis‐derived pluripotent stem cells [16, 17, 18].

While the effects of melatonin on cellular senescence markers like p16 and Oct4 have been documented, the precise molecular mechanisms by which melatonin regulates the expression of these markers remain unclear. Specifically, the mechanisms through which melatonin affects the nuclear functions of p16 and Oct4, as well as the potential interactions between these two markers, have not been fully explored. This study aims to elucidate the molecular mechanisms by which melatonin regulates p16 and Oct4 expression, focusing on the role of the newly identified nuclear receptor, vitamin D receptor (VDR), in mediating these effects.

## 2 Methods

### 2.1 Experimental Materials and Reagents

α‐MEM medium (high sugar) was purchased from Yingwei Jieji (Shanghai). 10% fetal bovine serum (FBS) was purchased from Beijing Quanshijin Biotechnology. Melatonin was purchased from Sigma‐Aldrich (Shanghai) Trading. Primary antibodies for p16 (1:1000 dilution; Cat. No. #23200), Oct4 (1:1000 dilution; Cat. No. #75463), GAPDH (1:1000 dilution; Cat. No. #2118), and Tubulin (1:1000 dilution; Cat. No. #2144) were obtained from Cell Signalling Technology. HRP‐labelled anti‐rabbit secondary antibody was purchased from Abcam (UK). LB broth, ampicillin, kanamycin, and other reagents were sourced from Qingdao Shengquan Chemical Reagents.

### 2.2 Cell Culture

The MC3T3‐E1 mouse osteoblast precursor cell line and bone marrow‐derived mesenchymal stem cells (BMSCs) were cultured in high‐glucose a‐MEM medium containing 10% FBS at 37°C in a 5% CO_2_ incubator. The cells were routinely passaged every 3–4 days and were sub‐cultured at a confluence of approximately 70%–80% for subsequent experiments.

### 2.3 Plasmid Construction

Primers were designed for the target genes, and PCR amplification was performed. The p16, Oct4, and VDR genes were inserted into the pGEX6p‐1 vector with an N‐terminal GST tag and into the pET28b vector with a 6 × His‐sumo tag. Plasmids were extracted using the SPARkeasy Ultra‐pure Plasmid Kit (SparkJade Science, China), and the sequences were verified by Sanger sequencing.

### 2.4 Protein Expression and Purification

For recombinant protein expression, E. coli was transformed with the plasmids and induced with IPTG at 16°C for 16–18 h. Cells were harvested and lysed using an ultrasonic cell disruptor. The supernatant was incubated with Ni‐NTA agarose beads for His‐Sumo tagged proteins or Glutathione Agarose 4B beads for GST‐tagged proteins at 4°C. After binding, the beads were washed with lysis buffer. ULP1 protease was used to cleave the His‐Sumo tag, and 3C protease was used for the GST tag. The purified proteins were further separated by gel filtration chromatography (Superdex 75 or Superdex 200 columns, GE Healthcare).

### 2.5 Isothermal Titration Calorimetry (ITC)

For interaction studies, purified proteins (p16, OCT4, and VDR) were dialyzed against a buffer containing 25 mM Tris–HCl and 150 mM NaCl (pH 8.0) to ensure buffer consistency and minimize heat of dilution. All samples were equilibrated in the same buffer prior to analysis. Isothermal titration calorimetry (ITC) measurements were performed using a MicroCal ITC200 system (Malvern Panalytical, UK) at 25°C. The protein solution (0.1 mM) was loaded into the sample cell, while melatonin (1 mM) was placed in the injection syringe. A series of 19 injections (2 μL per injection) were carried out with an interval of 150 s between injections under continuous stirring at 750 rpm. The initial injection (0.4 μL) was excluded from data analysis. Control titrations of melatonin into buffer were performed to correct for background heat effects. All solutions were degassed prior to loading to prevent air bubble formation.

The raw ITC data were integrated and analysed using Origin 7.0 software (OriginLab, USA). Binding isotherms were fitted using a one‐site binding model to determine the equilibrium dissociation constant (K_d). All experiments were independently repeated at least three times.

### 2.6 Western Blot Analysis

MC3T3‐E1 cells were treated with 0.2% DMSO, 10 nM, and 100 nM melatonin (dissolved in 0.2% DMSO) for 12 h. Total protein was extracted using RIPA lysis buffer containing protease inhibitors. Protein concentrations were measured using the BCA protein assay kit. Equal amounts of protein (20–30 μg) were separated by SDS‐PAGE and transferred to PVDF membranes. The membranes were blocked with 5% non‐fat dry milk in TBST for 2 h at room temperature. Membranes were incubated with primary antibodies (p16, Oct4, Tubulin) overnight at 4°C, followed by incubation with HRP‐conjugated secondary antibodies for 1 h at room temperature. Membranes were washed with TBST, and protein bands were detected using the ECL detection kit. Protein band intensity was quantified using Quantity One software (Bio‐Rad).

### 2.7 Real‐Time Quantitative PCR (RT‐qPCR)

Total RNA was extracted from the treated cells using TRIzol reagent (Invitrogen). cDNA was synthesized using the PrimeScript RT Reagent Kit (TaKaRa). RT‐qPCR was performed with the SYBR Premix Ex Taq II kit (TaKaRa) on an ABI Prism 7900HT system (Applied Biosystems). Tubulin was used as an internal reference gene. PCR amplification conditions were: 95°C for 30 s, followed by 95°C for 5 s and 60°C for 30 s for 40 cycles. Gene expression was calculated using the ΔΔCt method.

### 2.8 Statistical Analysis

All data are presented as mean ± standard deviation (SD). Two‐tailed Student's *t*‐test was used to compare two groups. *p* < 0.05 was considered statistically significant. All statistical analyses were performed using GraphPad Prism 8.0 (GraphPad Software).

## 3 Results

### 3.1 Expression and Purification of p16, Oct4, and VDR Proteins

**FIGURE 1:**
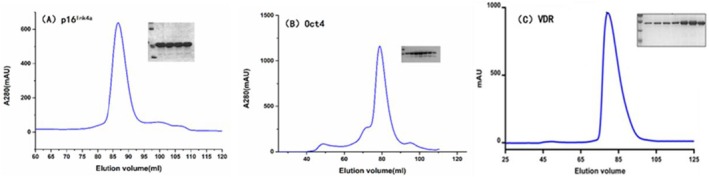
Recombinant p16 (A), Oct4 (B) and VDR (C) proteins were successfully expressed and purified from *E. coli* using GST and His‐Sumo labeling systems.

Recombinant p16, Oct4, and VDR proteins were successfully expressed and purified from *E. coli* using the GST and His‐Sumo tag systems. Following optimization of expression conditions, the proteins were purified to high homogeneity, with the purity confirmed by SDS‐PAGE analysis (Figure 1). Protein concentrations were determined by the Bradford assay, and the final purified proteins were used for subsequent experiments.

### 3.2 Melatonin Downregulates p16 and Upregulates Oct4 Expression

**FIGURE 2:**
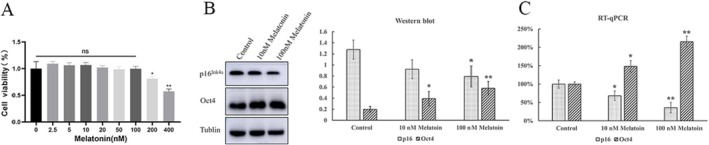
Effects of melatonin on cell viability and p16/Oct4 expression. (A) Cell viability after treatment with different concentrations of melatonin. (B) Western blot and quantitative analysis of p16^INK4a^ and Oct4 protein levels. (C) RT‐qPCR analysis of p16 and Oct4 mRNA expression. Data are presented as mean ± SD. *p < 0.05, **p < 0.01; ns, not significant.

To evaluate the effects of melatonin on cell viability and the expression of senescence‐associated markers, MC3T3‐E1 cells were treated with 0.2% DMSO, 10, or 100 nM melatonin for 12 h. CCK‐8 assays showed that melatonin treatment at the indicated concentrations did not significantly affect cell viability, indicating the absence of cytotoxic effects under these conditions (Figure 2A). Western blot analysis revealed that melatonin markedly decreased p16 protein levels in a dose‐dependent manner, while OCT4 protein expression was significantly increased following melatonin treatment (Figure 2B). Consistent with these results, RT‐qPCR analysis demonstrated a significant reduction in p16 mRNA expression and a concomitant increase in OCT4 mRNA levels in response to melatonin (Figure 2C). These findings indicate that melatonin modulates the expression of key senescence‐ and stemness‐associated markers without affecting cell viability, suggesting a regulatory rather than cytotoxic effect.

### 3.3 Interaction Between p16 and Oct4, and the Lack of Direct Interaction With Melatonin

**FIGURE 3:**
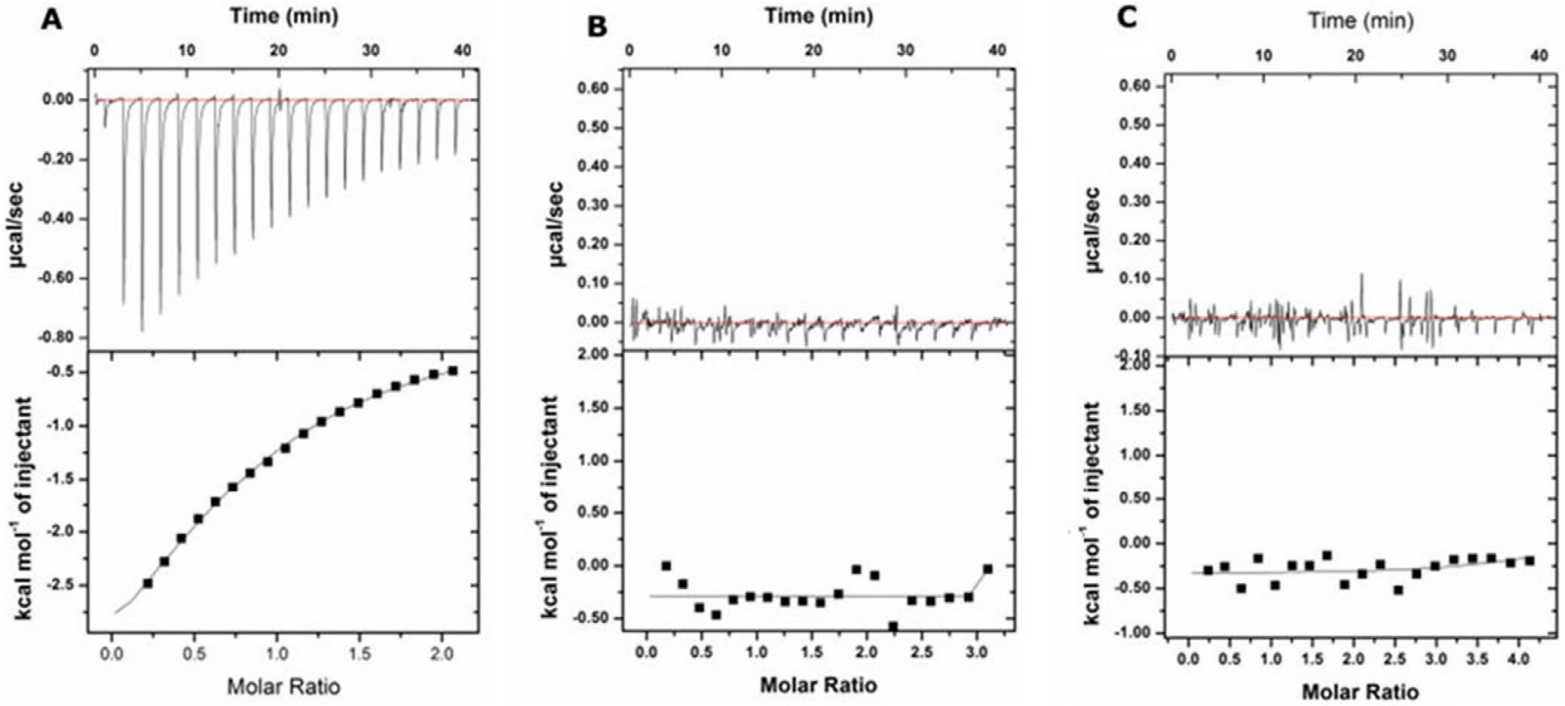
ITC analysis of interactions among p16, Oct4, and melatonin. (A) Direct binding of p16 to Oct4. (B) No detectable direct binding between melatonin and p16. (C) No detectable direct binding between melatonin and Oct4.

To examine the interaction between p16 and Oct4, ITC experiments were performed. The results showed a direct interaction between p16 and Oct4, with a dissociation constant (Kd) of approximately 70 μM (Figure 3A). In contrast, no direct binding was observed between melatonin and either p16 or Oct4, as demonstrated by ITC titrations of 1 mM melatonin into 0.1 mM p16 or 0.1 mM Oct4 (Figure 3B,C). These findings suggest that melatonin indirectly regulates the expression of these proteins without directly interacting with them.

### 3.4 Melatonin Regulates p16 and Oct4 Expression via VDR

**FIGURE 4:**
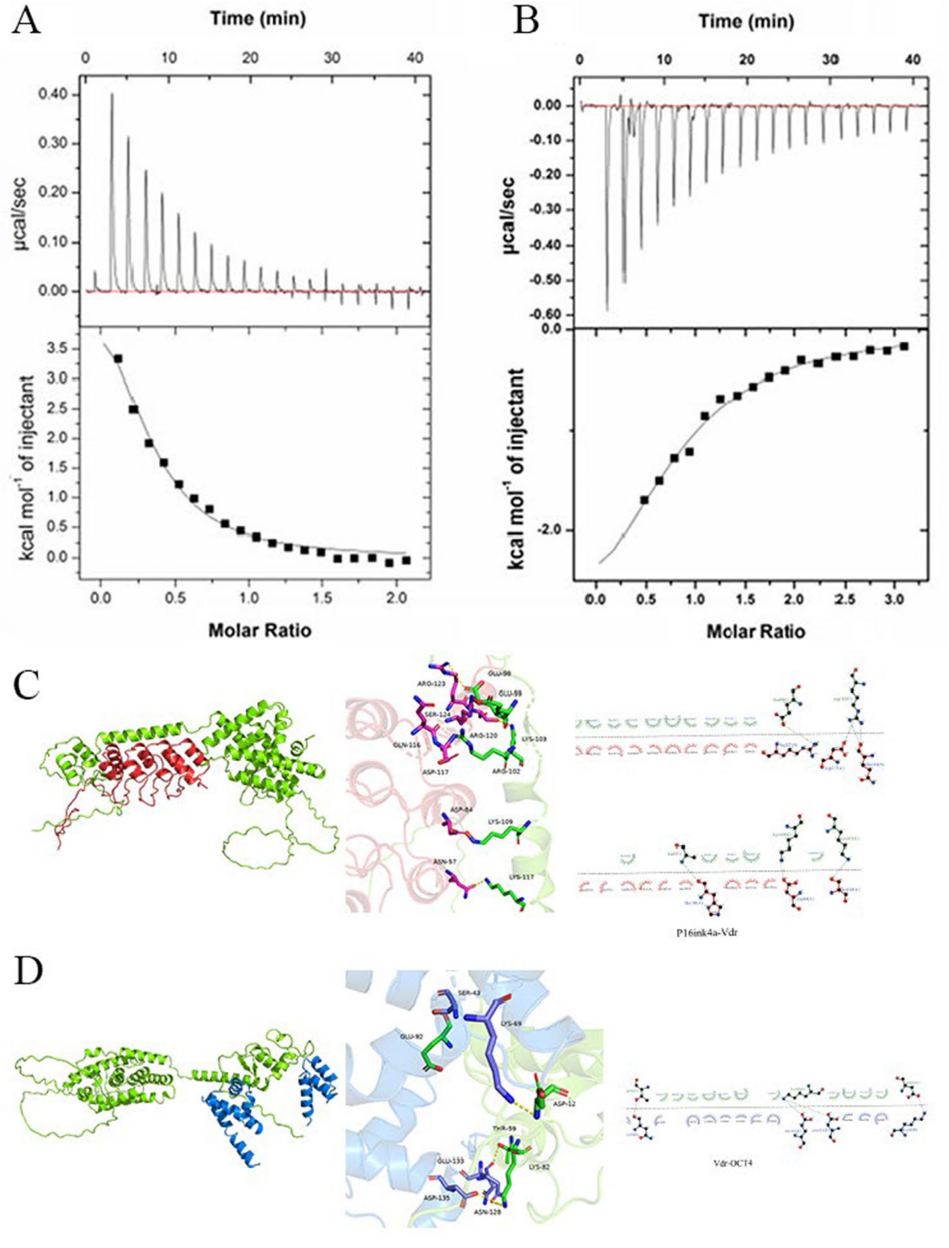
Interactions of VDR with p16 and OCT4. (A) ITC analysis of p16–VDR binding . (B) ITC analysis of OCT4–VDR binding. (C) Molecular docking models of p16–VDR and OCT4–VDR interactions. (D) Key amino acid residues involved in the interaction interfaces.

Given that melatonin is known to bind to the Vitamin D receptor (VDR), we next investigated the potential involvement of VDR in regulating p16 and OCT4. Isothermal titration calorimetry (ITC) analysis demonstrated that both p16 and OCT4 interact with VDR, as evidenced by the characteristic heat change profiles and saturable binding curves (Figure 4A,B). The calculated dissociation constants were approximately 30 μM for p16–VDR and 40 μM for OCT4–VDR, indicating moderate binding affinity. To further elucidate the structural basis of these interactions, molecular docking analysis was performed. The docking results showed that both p16 and OCT4 could be accommodated within defined binding pockets of VDR, forming stable complexes (Figure 4C). These interactions were primarily mediated by multiple non‐covalent forces, including hydrogen bonding and hydrophobic interactions, as illustrated by the three‐dimensional and two‐dimensional interaction maps (Figure 4C). Detailed analysis of the binding interfaces further identified key amino acid residues involved in stabilizing the complexes, providing structural support for the observed thermodynamic interactions (Figure 4D). Collectively, these findings suggest that VDR may function as a central interaction hub linking melatonin signalling to the regulation of p16 and OCT4.

### 3.5 Melatonin Regulates Osteogenic Differentiation Through a VDR‐Dependent Modulation of p16 and OCT4

**FIGURE 5:**
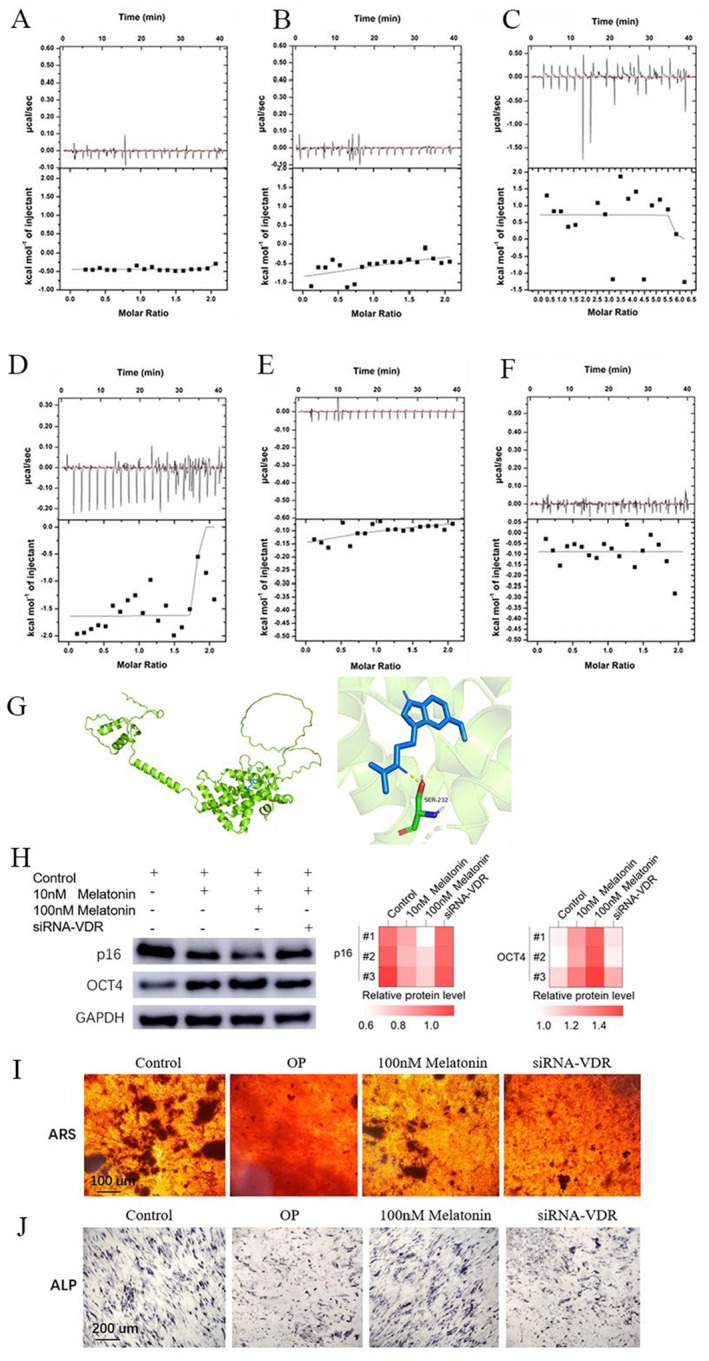
Melatonin regulates the VDR–p16/OCT4 axis and osteogenic differentiation. (A–F) ITC analyses showing the effects of melatonin on the interactions of VDR with p16 and OCT4. (G) Molecular docking analysis of melatonin with VDR and the predicted binding site. (H) Western blot analysis of p16 and OCT4 expression following melatonin treatment and VDR knockdown, with corresponding quantification. (I) Alizarin Red S (ARS) staining of BMSCs in the indicated groups. (J) Alkaline phosphatase (ALP) staining of BMSCs in the indicated groups.

To further investigate whether melatonin modulates the interaction between VDR and its associated proteins, we examined the effects of melatonin on the binding of OCT4 and p16 to VDR. The results indicated that melatonin enhanced the association between VDR and OCT4 while attenuating the interaction between VDR and p16 (Figure 5A), suggesting a differential regulatory effect on these protein complexes. To validate the functional role of VDR in this process, VDR expression was silenced, followed by Western blot analysis. The results showed that VDR knockdown significantly altered the expression levels of OCT4 and p16 (Figure 5B), indicating that VDR is required for maintaining their regulatory balance. To further assess the biological consequences of VDR‐mediated regulation, an osteoporosis‐related model was established using bone marrow‐derived mesenchymal stem cells (BMSCs). Osteogenic differentiation was evaluated by Alizarin Red S (ARS) staining and alkaline phosphatase (ALP) assays. The results demonstrated that VDR knockdown markedly impaired osteogenic differentiation, as evidenced by reduced mineralized nodule formation and decreased ALP activity (Figure 5C,D). In addition, molecular docking analysis revealed that both OCT4 and p16 could be accommodated within defined binding pockets of VDR, forming stable complexes through multiple non‐covalent interactions, including hydrogen bonding and hydrophobic contacts (Figure 5E). Detailed interaction mapping further identified key amino acid residues involved in stabilizing these complexes (Figure 5F), providing structural support for the observed interaction patterns. Collectively, these findings suggest that melatonin regulates osteogenic differentiation, at least in part, through a VDR‐centered regulatory axis involving differential modulation of OCT4 and p16 interactions.

## 4 Discussion

In this study, we systematically investigated the molecular mechanism by which melatonin regulates cellular senescence–associated pathways and identified a previously underappreciated melatonin–VDR–p16/Oct4 regulatory axis. Our findings establish a multi‐level mechanism in which melatonin does not directly interact with p16 or OCT4, but instead exerts its regulatory effects through the nuclear receptor VDR, which serves as a central interaction hub linking senescence and stemness pathways.

### 4.1 Melatonin and Cellular Senescence

Melatonin is a lipophilic indoleamine primarily synthesized by the pineal gland and is involved in regulating circadian rhythms, oxidative stress, and cellular homeostasis [7]. Numerous studies have highlighted melatonin's potential in mitigating the effects of aging, including its role in reducing cellular senescence and promoting tissue regeneration [4, 5, 6, 7]. In our study, we confirmed that melatonin downregulates p16, a key marker of cellular senescence, and upregulates Oct4, a gene associated with cellular reprogramming and pluripotency. This aligns with previous studies suggesting that melatonin may have a protective role against cellular aging by modulating cellular pathways involved in cell cycle regulation and stress responses [9, 10, 11].

### 4.2 Regulation of p16 and Oct4 Expression by Melatonin

Our results show that melatonin treatment significantly reduces the expression of p16 while simultaneously increasing Oct4 expression in the MC3T3‐E1 osteoblast precursor cell line. These findings are consistent with previous reports demonstrating that melatonin can modulate cellular senescence and extend the lifespan of various cell types, including stem cells [12, 13]. Although the mechanism by which melatonin regulates p16 and Oct4 was unclear prior to this study, our data suggest that this regulation occurs indirectly, as melatonin does not directly bind to either protein.

### 4.3 The Role of VDR in Melatonin's Effects

The key novel finding of this study is the identification of VDR as a mediator of melatonin's effects on p16 and Oct4 expression. VDR, a member of the nuclear receptor superfamily, is involved in the regulation of gene expression in response to vitamin D. Recent studies have shown that melatonin can bind to VDR, leading to the formation of a melatonin‐VDR complex that modulates gene expression in the nucleus [16]. Our ITC data support this finding, as VDR directly interacts with both p16 and Oct4. The observed Kd values suggest that this interaction is physiologically relevant, and VDR likely acts as a mediator for melatonin's nuclear entry, which then facilitates the regulation of these key senescence‐related proteins. These results suggest that the VDR pathway is a critical component of the mechanism by which melatonin regulates cellular senescence.

### 4.4 Implications for Cellular Reprogramming and Aging

Our findings support that melatonin may play a central role in cellular reprogramming and anti‐aging therapies. Oct4, a pivotal factor in cellular reprogramming, is known to maintain pluripotency and self‐renewal in stem cells. The ability of melatonin to upregulate Oct4 while simultaneously downregulating p16 provides a potential mechanism [19] through which melatonin could enhance cellular rejuvenation and extend the functional lifespan of cells. This has significant implications for regenerative medicine and the treatment of age‐related disorders, particularly in the context of stem cell‐based therapies [20, 21]. Moreover, the interaction between melatonin and VDR may offer new insights into the development of therapeutic strategies that target the VDR pathway to mitigate age‐related diseases. Given the established role of VDR in regulating bone metabolism and immune function, targeting the VDR‐melatonin axis could have broad therapeutic applications for both aging and osteoporosis, conditions that are often characterized by accelerated cellular senescence and loss of tissue function.

While our study provides valuable insights into the regulatory mechanisms underlying melatonin's anti‐senescence effects, further research is needed to confirm these findings in vivo [22]. From a signalling perspective, melatonin has been traditionally understood to exert its effects primarily through membrane receptors such as MT1 and MT2. However, our findings highlight a complementary and potentially distinct role of VDR as a nuclear mediator of melatonin signalling. Unlike MT1/MT2, which mainly mediate rapid signalling responses, VDR functions as a transcriptional regulator that controls long‐term gene expression programs. Therefore, the melatonin–VDR axis identified in this study may represent a nuclear signalling pathway that complements classical membrane receptor–mediated mechanisms, particularly in the context of cellular senescence and differentiation.

Despite these strengths, several limitations of this study should be acknowledged. First, although we demonstrated the functional involvement of VDR using knockdown experiments, more rigorous causal validation, such as rescue experiments or genetic knockout models, would further strengthen the mechanistic conclusions. Second, cellular senescence in this study was primarily inferred from the expression of p16 and OCT4, and more direct functional assays, such as SA‐β‐gal staining or cell cycle analysis, were not performed. Third, although we incorporated BMSC‐based osteogenic assays to enhance physiological relevance, in vivo validation in animal models remains necessary to confirm the role of the melatonin–VDR axis in aging‐related diseases such as osteoporosis. Finally, while molecular docking supports the structural basis of protein interactions, high‐resolution structural or biochemical validation (e.g., co‐immunoprecipitation or mutagenesis studies) would provide more definitive evidence.

In conclusion, this study identifies VDR as a critical mediator linking melatonin signalling to the coordinated regulation of senescence and stemness pathways. By integrating thermodynamic, structural, and functional evidence, we propose a novel VDR‐centered regulatory axis that may contribute to the anti‐senescence and pro‐regenerative effects of melatonin. These findings provide new insights into the molecular basis of melatonin action and suggest potential therapeutic strategies targeting the VDR pathway in aging and age‐related diseases.

## 5 Conclusion

This study demonstrates that melatonin regulates cellular senescence by downregulating p16 and upregulating Oct4 expression through the nuclear receptor VDR. These findings suggest that melatonin may play a crucial role in counteracting cellular aging and promoting tissue regeneration. Further in vivo studies are needed to confirm the physiological relevance and therapeutic potential of these effects.

## Author Contributions

**Yang Xu:** conceptualization (equal), investigation (equal), methodology (equal), writing – original draft (equal). **Cong Zhang:** conceptualization (equal), formal analysis (equal), investigation (equal), software (equal). **Li Wang:** conceptualization (equal), investigation (equal), methodology (equal), project administration (equal), validation (equal). **Xiaoyu Chen:** project administration (equal), resources (equal), writing – review and editing (equal).

## Funding

The authors have nothing to report.

## Conflicts of Interest

The authors declare no conflicts of interest.

## Data Availability

The data available for this paper are included in the figures and tables.
